# N-acetylation and phosphorylation of Sec complex subunits in the ER membrane

**DOI:** 10.1186/1471-2121-13-34

**Published:** 2012-12-13

**Authors:** Christina Soromani, Naiyan Zeng, Klaus Hollemeyer, Elmar Heinzle, Marie-Christine Klein, Thomas Tretter, Matthew N J Seaman, Karin Römisch

**Affiliations:** 1 Department of Microbiology, Faculty of Biology, Saarland University, Campus A1.5, 66123, Saarbruecken, Germany; 2 CIMR, Cambridge, UK; 3 Department of Technical Biochemistry, Faculty of Chemistry, Saarland University, Saarland, Germany; 4 Shanghai Jiao-Tong University School of Medicine, Shanghai, China; 5 Department of Clinical Biochemistry, University College London Hospital, London, UK

**Keywords:** Protein translocation, Endoplasmic Reticulum, Sec complex, Sbh1p, Sec62p, Sec61p, Phosphorylation, N-acetylation, Convergent evolution, ER targeting

## Abstract

**Background:**

Covalent modifications of proteins provide a mechanism to control protein function. Here, we have investigated modifications of the heptameric Sec complex which is responsible for post-translational protein import into the endoplasmic reticulum (ER). It consists of the Sec61 complex (Sec61p, Sbh1p, Sss1p) which on its own mediates cotranslational protein import into the ER and the Sec63 complex (Sec63p, Sec62p, Sec71p, Sec72p). Little is known about the biogenesis and regulation of individual Sec complex subunits.

**Results:**

We show that Sbh1p when it is part of the Sec61 complex is phosphorylated on T5 which is flanked by proline residues. The phosphorylation site is conserved in mammalian Sec61ß, but only partially in birds, and not in other vertebrates or unicellular eukaryotes, suggesting convergent evolution. Mutation of T5 to A did not affect the ability of mutant Sbh1p to complement the growth defect in a *Δsbh1Δsbh2* strain, and did not result in a hypophosphorylated protein which shows that alternate sites can be used by the T5 kinase. A survey of yeast phosphoproteome data shows that Sbh1p can be phosphorylated on multiple sites which are organized in two patches, one at the N-terminus of its cytosolic domain, the other proximal to the transmembrane domain. Surprisingly, although N-acetylation has been shown to interfere with ER targeting, we found that both Sbh1p and Sec62p are cotranslationally N-acetylated by NatA, and N-acetyl-proteome data indicate that Sec61p is modified by the same enzyme. Mutation of the N-acetylation site, however, did not affect Sec62p function in posttranslational protein import into the ER. Disabling NatA resulted in growth retardation, but not in co- or posttranslational translocation defects or instability of Sec62p or Sbh1p.

**Conclusions:**

We conclude that N-acetylation of transmembrane and tail-anchored proteins does not interfere with their ER-targeting, and that Sbh1p phosphorylation on T5, which is not present in Sbh2p, plays a non-essential role specific to the Sec61 complex.

## Background

Protein phosphorylation is a reversible mechanism used in all kingdoms of life to regulate protein activity, location and stability [1]. Protein N-acetylation which is irreversible can regulate protein stability and protein-protein interactions [2,3]. Many proteins (50% in yeast) are N-acetylated, the enzymes responsible for N-acetylation have been identified, and their substrate specificities characterized [4]. The role of N-acetylation, however, remains unclear for the majority of substrates to date. Strikingly, proteins bearing N-terminal signal sequences are usually not N-acetylated [5]. If the modification is introduced by mutation, N-acetylation leads to missorting of secretory proteins to the cytosol. These observations led to the conclusion that N-acetylation interferes with ER targeting [5].

Although protein flux across the ER membrane can be extremely variable, nothing is known about the regulation of the activity of the protein translocation channel in the ER membrane. In yeast the channel is composed of 3 subunits, Sec61p, Sbh1p and Sss1p, which form the Sec61 complex responsible for cotranslational protein import into the ER [6]. The channel subunits are highly conserved with mammalian proteins Sec61α, Sec61ß and Sec61γ. In yeast, posttranslational import into the ER of proteins with less hydrophobic signal sequences is mediated by a heptameric complex which in addition to the Sec61 complex contains the Sec63 complex (Sec63p, Sec62p, Sec71p, Sec72p) [6]. Yeast also express a homologue of Sec61p, Ssh1p, which together with Sss1p and a homologue of Sbh1p, Sbh2p, forms the Ssh1 complex responsible exclusively for co-translational import into the ER [6]. Protein translocation into the ER and the *SEC61*, *SSS1*, *SEC63* and *SEC62* genes are essential. Deletion of either *SBH1* or *SBH2* does not affect yeast viability, but deletion of both genes leads to temperature-sensitivity [7].

Sbh1p and Sbh2p interact with multiple partners, and it is not known how these interactions are regulated. Sbh1p and Sbh2p are small tail-anchored proteins in the ER membrane with largely unstructured cytosolic domains and single α-helical transmembrane domains which on their own can complement the temperature sensitivity of a *Δsbh1Δsbh2* strain [8,9]. The cytosolic domain of Sbh2p, however, modulates interactions of its transmembrane domain [10], and this is likely also the case for the Sbh1p cytosolic domain. Sbh2p is required for efficient transfer of the nascent polypeptide from signal recognition particle (SRP) into the Ssh1 channel, and may signal to the SRP receptor whether the Ssh1 channel is already occupied by a ribosome [10]. In mammalian cells, Sec61ß mediates association of signal peptidase with the Sec61 channel [11]. The cytosolic domain of Sec61ß can bind to the ribosome, and can be crosslinked to nascent secretory proteins within the ribosomal exit tunnel [12,13], but the principal ribosome receptor in the ER membranen is Sec61p [14,15]. The cytosolic domain of Sec61ß also serves as GDP-exchange factor for the ß subunit of SRP receptor in the ER membrane [16]. In addition to its function in translocation, *SBH1* also interacts both genetically and physically with the exocyst, a protein complex required for fusion of secretory transport vesicles with the plasma membrane [17]. This function is specific to Sbh1p; overexpression of Sbh2p, which is 50% identical to Sbh1p at the amino acid level, does not suppress exocyst mutations [17]. Mammalian Sec61ß also binds to the exocyst [18]. Sbh1p interaction with the exocyst requires its cytosolic domain, but the function of the interaction remains unknown [8].

Mammalian Sec61ß has been shown to be phosphorylated [19]. In isolated ER membranes phosphorylation is mediated by Protein Kinase C, and phosphorylation of ER-derived microsomes enhances cotranslational protein translocation into these membranes. It was not shown, however, that Sec61ß was the protein whose phosphorylation was responsible for enhanced translocation. Sec61ß was also found to be phosphorylated in intact cells, but the kinase and the site(s) of phosphorylation were not identified. The only other subunit of the Sec complex which is known to be phosphorylated is Sec63p [20]. Phosphorylation enhances association of Sec63p with Sec62p by increasing acidity of the carboxy-terminal region of Sec63p, and is mediated by casein kinase 2 (CK2). Whether Sec63p phosphorylation has a regulatory function and whether mammalian Sec63p is also phosphorylated is unknown so far.

Here we set out to investigate the role of phosphorylation in Sbh1p function. In purified Sec complexes we identified the threonine at position 5 as a phosphorylated residue. Mutation of T5 to alanine, however, did not affect the ability of Sbh1p to complement the temperature-sensitivity of a *Δsbh1Δsbh2* strain, and did not result in detectable hypophosphorylation of the cellular pool of the protein. Comparison to multiple phosphoproteome data revealed additional phosphorylation sites in Sbh1p which were modified in several combinations. Analyzing the entire Sec complex by mass spectrometry we were surprised to find that both Sbh1p and Sec62p were N-acetylated although this modification has been shown to inhibit targeting to the ER [5]. Mutation of the N-acetyl acceptor site in Sec62p did not affect post-translational protein import into the ER. A strain deleted for the enzymatically active subunit of the NatA complex, Ard1p (also known as Naa10p), which is responsible for N-acetylation of Sec62p and Sbh1p, showed growth defects that were exacerbated at low and high temperature. The strain, however, displayed no measurable protein translocation defects, and both Sec62p and Sbh1p were stably expressed in the *Δard1* strain, suggesting that N-acetylation of these proteins by Nat A is not essential for protein import into the ER.

## Results

### Sbh1p can be phosphorylated at the ER membrane

**Figure 1:**
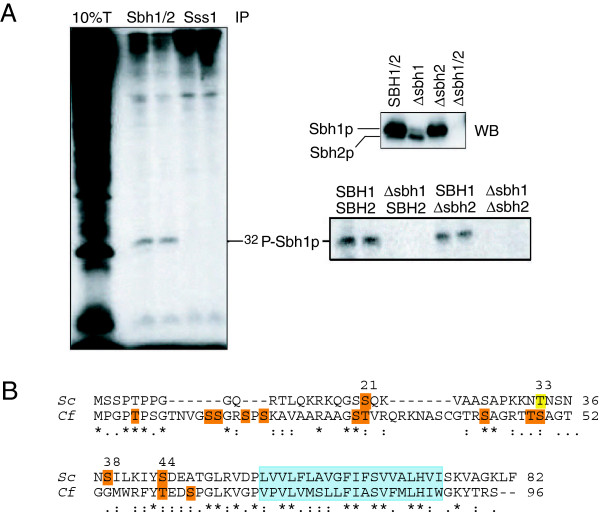
**Sbh1p can be phosphorylated in yeast ER membranes.** (**A**) Left: Wildtype microsomes (5eq) were incubated with g-[^32^P]ATP (40 μCi) for 30 min. Membranes were lysed in SDS, and Sbh1p and Sss1p immunoprecipitated with polyclonal rabbit antisera in duplicate. Precipitated proteins and 10% of the lysate were resolved on a 15% SDS gel and detected by autoradiography. Upper right: Microsomes (1 eq) of the indicated strains were lysed, proteins resolved on a 15% SDS gel, and Sbh proteins detected with an antiserum directed against the first 18 amino acids of Sbh1p. Lower right: Microsomes from the indicated strains were labelled with γ-[^32^P]ATP as above and Sbh proteins precipitated in duplicate. Proteins were resolved on a 15% gel and detected by autoradiography. (**B**) Alignment of *S. cerevisiae* Sbh1p and *Canis familiaris* Sec61ß. Transmembrane domains are indicated in blue. Phosphorylation sites predicted by NetPhos are shown in orange, an additional site predicted by Scan Prosite in yellow.

When dog pancreas microsomes are incubated with γ-^32^P]ATP ER-associated protein kinase C phosphorylates mammalian Sec61ß [19]. We asked whether the yeast orthologue of Sec61ß, Sbh1p, could similarly be phosphorylated in a cell-free system. We prepared microsomes from a wildtype yeast strain and incubated 5eq membranes in the presence of phosphatase inhibitors, GTP, and calcium with 40 μCi γ-^32^P]ATP for 30 min at 30°C. Membranes were lysed in SDS and Sbh1p and Sss1p immunoprecipitated. As shown in Figure 1A, the Sbh1p antibody, but not the Sss1p antibody, precipitated a radiolabelled protein from the lysed microsomes. The Sbh1p antibody was raised against the first 18 amino acids of Sbh1p. On Western Blots it also recognizes the Sbh2 protein (Figure 1A, upper right panel), but in the immunoprecipitation we detected a single phosphate-labelled protein (Figure 1A, left panel). In order to investigate whether this protein corresponded to Sbh1p or Sbh2p, we prepared microsomes from strains in which either the *SBH1* or the *SBH2* gene had been deleted, labelled the microsomes with γ-^32^P]ATP as above, and immunoprecipitated Sbh proteins. As shown in Figure 1A (lower right panel) we could only detect phosphate-labelled Sbh1p in our membranes. We conclude that Sbh1p, like Sec61ß in mammalian membranes, can be phoshorylated at the ER membrane.

### Mutation of predicted Sbh1p phosphorylation sites S21, S38, S44 & T33

**Figure 2:**
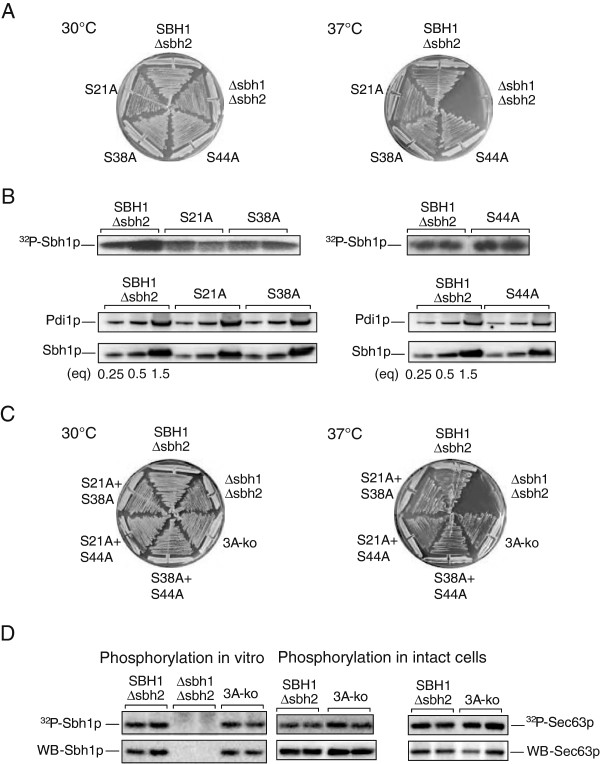
**Mutation of the predicted phosphorylation sites in Sbh1p.** (**A**) The indicated sites were mutated to alanine, and mutants expressed in a *Δsbh1Δsbh2* strain. *SBH1Δsbh2* yeast were used as a control. Strains were grown on YPD plates at 30°C and 37°C for 2d. (**B**) Upper: Membranes from the indicated strains were labelled with gamma γ-[^32^P]ATP and Sbh1 proteins analyzed as above. Lower: The indicated amount of microsomes was dissolved in SDS sample buffer, proteins separated by gel electrophoresis and Sbh1p and Pdi1p (as a loading control) detected by immunoblotting. (**C**) As in (A). (**D**) Sbh1 mutant proteins from strains shown in (**C**) were either labelled with g-[^32^P]ATP in microsomes as above (left panel), or labelled in intact cells with [^32^P]phosphate; proteins were immunoprecipitated, separated on 15% SDS gels, and detected by autoradiography. Sec63p was used as a control phosphoprotein. Immunoprecipitated proteins were also detected by immunoblotting (lower panels).

Gruss and colleagues [19] had not identified the sites phosphorylated in dog Sec61ß. We looked for potential phosphorylation sites in Sbh1p using the NetPhos programme. We found three phosphorylation sites in the cytosolic domain of Sbh1p (shown in orange in Figure 1B), two of which were conserved in dog Sec61ß. Yeast strains which lack both *SBH1* and *SBH2* are temperature-sensitive at 37°C, but yeast tolerate deletion of either gene without measurable translocation defects [17]. We asked whether mutation of the potential phosphorylation sites in *SBH1* affected the ability of the gene to complement the growth defect of a *Δsbh1Δsbh2* strain at 37°C. Amino acids S21, S38, and S44 in Sbh1p were changed to alanine using site-directed mutagenesis and the resulting genes expressed in *Δsbh1Δsbh2* yeast. As shown in Figure 2A, all mutants were still able to promote growth of this strain at 37°C. Overexpression of all mutants also still rescued exocyst mutants *sec8-9* and *sec15-1* (not shown). We then asked whether the mutant proteins were still phosphorylatable. Membranes were prepared from each strain, labelled with γ-^32^P]ATP, and Sbh1p was immunoprecipitated. As shown in Figure 2B (upper panels) all mutant proteins were still phosphorylated to levels comparable with wildtype protein. A constant phospho-protein signal could have been the result of overexpression of hyposphosphorylated Sbh1p. Immunoblotting on the membranes, however, revealed that expression levels of wildtype and the individual mutant proteins were comparable (Figure 2B, lower panels). We conclude that none of the identified potential phosphorylation sites (S21, S38, S44) in the cytosolic domain of Sbh1p is the sole phosphate-acceptor site in the protein.

We then combined the mutations of the individual potential phosphorylation sites in pairs or in a triple alanine mutant. As shown in Figure 2C even the triple alanine mutant still complemented growth of *Δsbh1Δsbh2* cells at high temperature. The mutant protein could still be phosphorylated at the ER membrane in vitro to levels comparable with wildtype Sbh1p (Figure 2D, left panel). [^32^P]Phosphate-labelling of intact yeast cells also revealed that tripleA-Sbh1p was phosphorylated like wildtype Sbh1p, and expressed at the same level (Figure 2D, middle panel). Sec63p, a known phosphoprotein subunit of the Sec complex, served as a control in this experiment (Figure 2D, right panel). We conclude that S21, S38, and S44 are not the phosphate acceptor sites, or not the only phosphate acceptor sites in Sbh1p.

When we used a different prediction programme, Scan ProSite, we were able to identify an additional phosphorylation site in the cytosolic domain of Sbh1p at T33 (Figure 1B, yellow). Mutation of T33 to alanine on its own or in combination with S21A, S38A and S44A, however, did not affect complementation activity of the mutant Sbh1p, nor its phosphorylation level (not shown).

### Identification of T5 as phospho-acceptor site in Sbh1p by mass spectrometry

**Figure 3:**
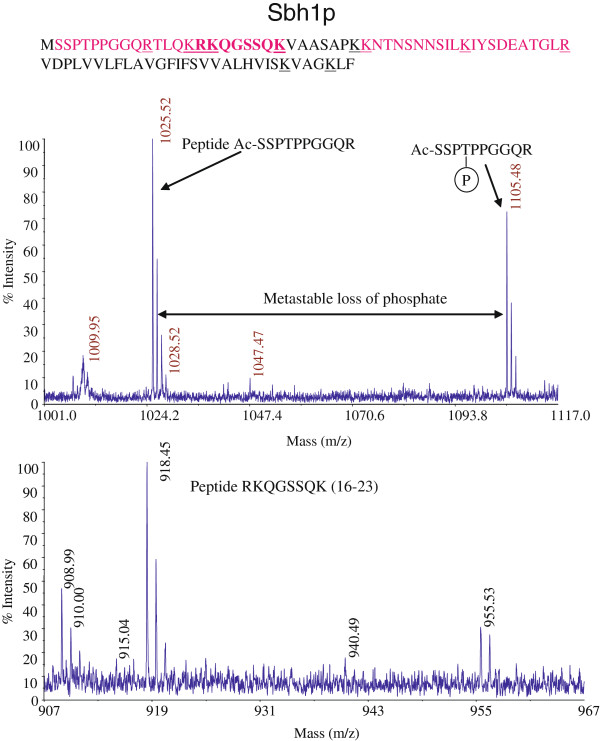
**Identification of a phosphorylated site in Sbh1p by mass spectrometry.** Sec complex was purified using membranes from an *SSS1-HA* tagged strain as in Methods. The Sbh1p band was excised, digested with trypsin, and the resulting peptides analyzed by mass spectrometry. The amino acid sequence of Sbh1p is shown. Peptides covered in the analysis are shown in pink. Trypsin cleavage sites are underlined. Peaks corresponding to the phosphorylated peptide are indicated. Note that S27 is the only potential phosphate acceptor site in the Sbh1p cytosolic domain not covered by this analysis.

We then purified the Sec complex from ER membrane to analyze Sbh1p posttranslational modifications by mass spectrometry. Microsomes (7500eq) from a strain in which Sss1p was tagged with the HA-epitope were lysed in digitonin, the resulting lysate subjected to ultracentrifugation for 1 h at 200,000 g to remove membrane debris and ribosomes, and Sss1p-HA and associated proteins were purified by affinity chromatography. Proteins were resolved by SDS-PAGE on a 15% gel and the band corresponding to Sbh1p was excised and analyzed by mass spectrometry. As shown in Figure 3 we were able to identify a single phosphopeptide in the cytosolic domain of Sbh1p with the phosphate attached to T5. With the exception of a small peptide including S27 (Figure 3, black letters) our analysis covered the entire cytosolic domain of Sbh1p. We repeated the purification, but were still unable to get data from the peptide including S27. We conclude that Sbh1p when associated with the Sec61 complex is phosphorylated on T5.

### Mutation of T5 does not interfere with Sbh1p function

**Figure 4:**
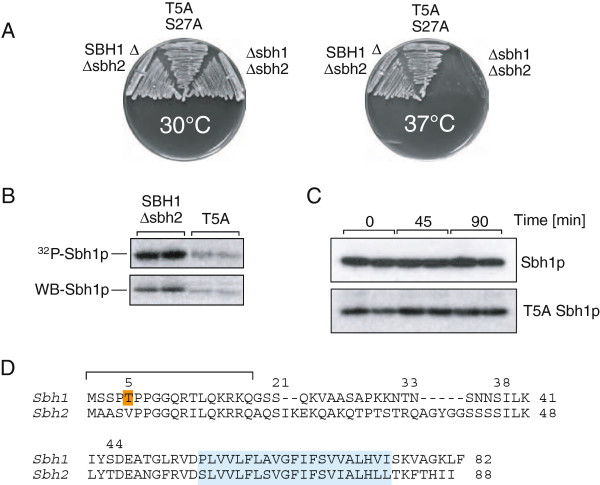
**Mutation of the known phosphate acceptor site in *SBH1.*** (**A**) The indicated sites were mutated to alanine, and mutant Sbh1 proteins expressed in a *Δsbh1Δsbh2* strain. The *SBH1Δsbh2* strain was used as a control. Strains were grown on YPD plates at 30°C and 37°C for 2d. (**B**) Sbh1 mutant proteins from strains shown in (A) were labelled in intact cells with [^32^P]phosphate; cells were lysed and proteins immunoprecipitated, separated on 15% SDS gels, and detected by autoradiography. Immunoprecipitated proteins were also detected by immunoblotting (lower panels). (**C**) *SBH1Δsbh2* and *sbh1-T5AΔsbh2* strains were grown to early exponential phase, cycloheximide added to prevent further protein synthesis, samples taken at the indicated times, cells lysed, proteins resolved on SDS gels and Sbh1 proteins detected by immunoblotting. (**D**) Alignment of Sbh1p and Sbh2p. The identified phosphorylation site in Sbh1p is indicated in orange, transmembrane domains are shown in blue. The bracket denotes the peptide of Sbh1p against which our polyclonal antibody was raised.

Next we mutated Sbh1p T5 to alanine either alone or in combination with S27. Both the single (not shown) and the double mutant were still able to complement growth of the *Δsbh1Δsbh2* strain at high temperature (Figure 4A). In [^32^P]phosphate-labelling experiments, the T5A mutant showed a weaker signal in autoradiography (Figure 4B, upper), but the corresponding protein levels also were reduced (Figure 4B, lower), thus the mutation did not result in a hypophosphorylated protein. Adding the S27A mutation had no additional effects on phosphorylation or expression (not shown). This suggest that the kinase responsible for phosphorylating T5 is promiscuous and can use other residues in the vicinity, perhaps the serine at position 3 (Figure 4D).

The reduced signal in the Sbh1p Western Blot of the T5A mutant suggested that the T5A mutation destabilizes Sbh1p. This would not necessarily manifest itself in a functional defect, as *SBH2* expressed from its own promoter can complement the growth defect of *Δsbh1Δsbh2* yeast at 37°C although it is only expressed at about 10% of Sbh1p (Figure 1A, upper right). We performed a cycloheximide chase to investigate T5A Sbh1p stability. Cells were incubated with cycloheximide to prevent new protein synthesis, aliquots were taken at 0, 45 and 90 min, cells lysed and Sbh1p detected by immunoblotting. As shown in Figure 4C both wildtype and T5A Sbh1p were stable for the entire chase period.

Another potential explanation for the reduced signal in T5A Sbh1p Western Blots is that our antibody does not recognize the mutant protein as well as the wildtype. We raised the antibody against the first 18 amino acids of Sbh1p (Figure 4D, bracket). The antibody recognizes both Sbh1p and Sbh2p on blots (Figure 1A, upper right), but we were unable to precipitate phosphate-labelled Sbh2p (Figure 1A, lower right) or unlabelled Sbh2p (not shown) using the antibody, suggesting that it recognizes Sbh1p better than Sbh2p. Within the first 18 amino acids the only region that differs between the two proteins is amino acids 2–5 (Figure 4D). If this region is decisive for interaction with the antibody, mutation of T5 to A may result in the reduced signal that we see in immunoprecipitations of the phosphorylated mutant protein and since the blots shown in Figure 4B are on the immunoprecipitated material, this would also explain the reduced signal for T5A Sbh1p.

### Phosphorylation of Sbh1p is dynamic

**Figure 5:**
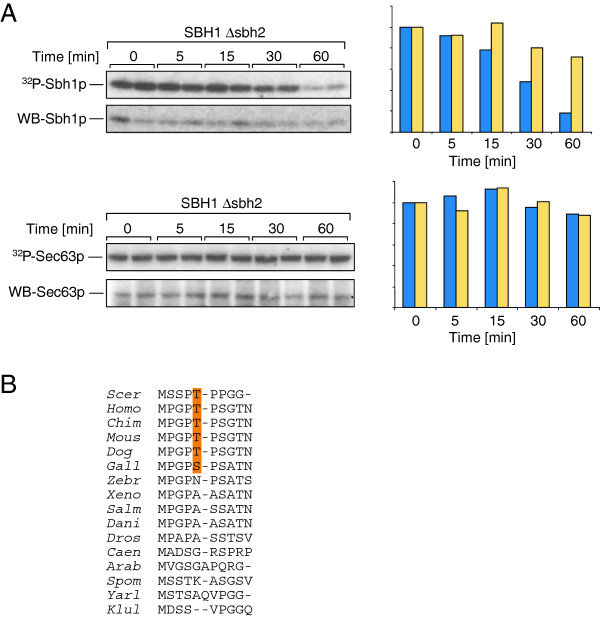
**Sbh1p phosphorylation is dynamic.** (**A**) *SBH1Δsbh2* cells were labelled with [^32^P]phosphate for 10 min and chased for the indicated times. Cells were lysed, Sbh1p and Sec63p immunoprecipitated, proteins resolved by SDS-PAGE and detected by autoradiography (upper panels) and immunoblotting (lower panels). Signals were quantified using a phosphorimager and relative amounts of protein (yellow) and phosphoprotein (blue) shown in the graphs. (**B**) N-termini of Sbh1p homologues were aligned.

Phosphorylation is often used to regulate protein activity or interaction with specific partners and is therefore usually reversible [1]. We asked whether phosphorylation of Sbh1p was transient. We labelled *SBH1Δsbh2* cells with ^32^P]phosphate for 10 min, chased for up to 60 min and immunoprecipitated Sbh1p from the lysed cells. We found that while Sbh1p itself was more or less stable over this period, the phosphate on the protein had a half live of approximately 30 min (Figure 5A, upper). As a control we immunoprecipitated another phosphorylated subunit of the Sec complex, Sec63p, and found that the phosphate on Sec63p was stable (Figure 5A, lower). We conclude that the phosphate on Sbh1p is turned over and is therefore likely to have a regulatory function.

We found that Sbh1p was phosphorylated on T5 (Figure 3). While *SBH1* homologues are present in all eukaryotes, the cytosolic domain of the protein is not highly conserved and there has been speculation that it might serve different functions in different organisms [21]. Upon sequence comparison, we found that T5 is conserved in mammals and *S. cerevisiae*, but not strictly in birds, and not at all in other vertebrates, flies, nematodes, plants, or other yeasts (Figure 5B). In all these organisms, however, there are phosphorylatable serine residues in close proximity, so a position close to the N-terminus could potentially be phosphorylated in all Sbh1p orthologues. Strikingly, the proline in position 4 is conserved yeast, all vertebrates, and even in *Drosophila*, whereas the proline in position 6, which is part of the recognition sequence for proline-directed kinases, is only present in the species that also have a phosphorylatable residues in position 5 (Figure 5B) [22].

### Sbh1p and Sec62p are N-acetylated

**Figure 6:**
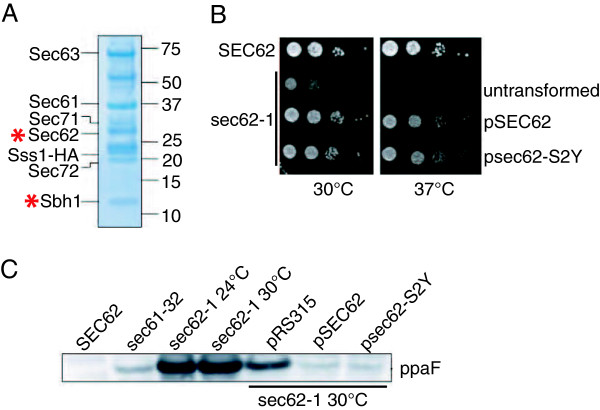
**Sec62p and Sbh1p are N-acetylated.** (**A**) Sec complexes and ribosome-free Sec61 complexes were purified from an *SSS1-HA* strain by affinity chromatography as described in Methods. Individual bands were excised, trypsin-digested and analyzed by mass spectrometry. N-acetylated proteins are indicated by red asterisks. (**B**) Mutant *sec62-1* cells were transformed with a plasmid expressing wildtype *SEC62* or N-acetylation acceptor site mutant *sec62-S2Y*. *SEC62* wildtype cells and untransformed and transformed sec62-1 cells were diluted and growth on solid media monitored at the indicated temperatures. (**C**) *SEC62* wildtype or the indicated mutant cells without or with the indicated plasmids were grown to early exponential phase, cells lysed, proteins separated by SDS-PAGE and prepro-alpha factor (ppaF) detected by immunoblotting.

Because N-acetylation of soluble secretory proteins has been shown to interfere with their targeting to the ER membrane [5], we were surprised to find that Sbh1p is also acetylated at its N-terminus (Figure 3). The effect of N-acetylation on membrane proteins in particular has not been explored so far. Since the Sec complex subunits are relatively long-lived, and the modification can contribute to protein stability [2], we asked whether other subunits of the complex were also N-acetylated. We repeated the purification of the Sec complex as above. This time, all bands corresponding to Sec complex subunits were excised, proteins digested with trypsin, and the resulting peptides analyzed by mass spectrometry. As shown in Figure 6A we were able to identify all subunits of the Sec complex. Of these, only Sbh1p and Sec62p were N-acetylated. Since Sec62p is an essential protein required for posttranslational protein import into the ER, we asked whether N-acetylation is important for its function. We mutated the serine in position 2 to tyrosine, which cannot be N-acetylated [23]. The Sec62-S2Y protein was able to complement the growth defect in the temperature-sensitive *sec62-1* mutant (Figure 6B). A sensitive marker for posttranslational protein import defects into the ER is the cytosolic accumulation of the precursor of the alpha factor pheromone, prepro-alpha factor (ppaF). The precursor is undetectable in wildtype cells (Figure 6C, SEC62) because it is rapidly translocated into the ER, transported to the Golgi and proteolytically cleaved. In translocation defective cells (Figure 6C, sec61-32, sec62-1) ppaF accumulates. Transformation of *sec62-1* cells with a plasmid containing wildtype *SEC62* alleviates the translocation defect (Figure 6C, pSEC62). Transformation with a plasmid containing the N-acetylation mutant *sec62-SY2* complements the translocation defect like the wildtype gene (Figure 6C). We conclude that N-acetylation is not essential for Sec62p function.

### Inactivation of NatA reduces growth, but not ER translocation

**Figure 7:**
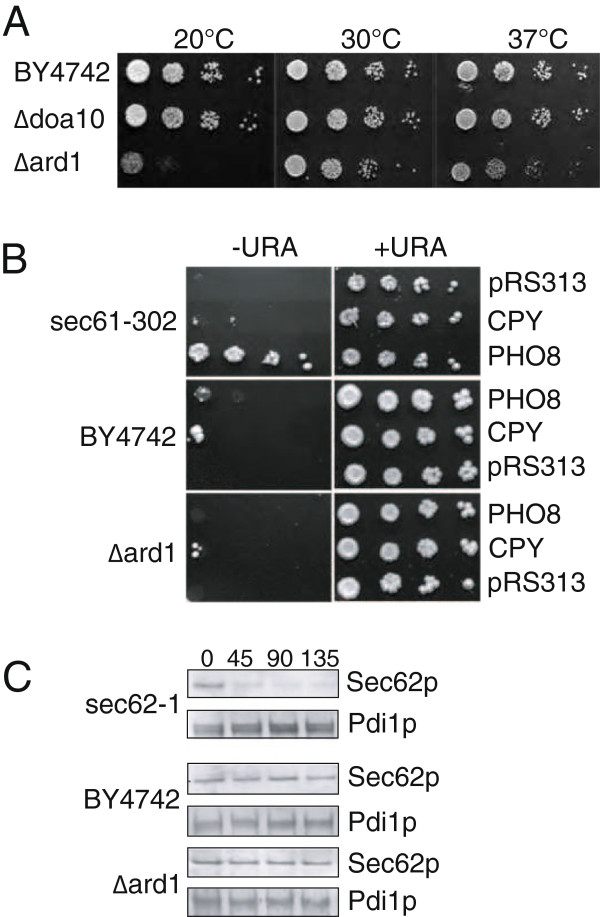
***Δard1 *yeast have a growth defect, but no translocation defect or Sec62p instability.** (**A**) Wildtype (BY4742) and the indicated mutant cells were diluted on YPD plates and growth monitored at the indicated temperatures. (**B**) Wildtype, *sec61-302* and *Δard1* cells were transformed with empty vector (pRS313), or plasmids expressing *PRC1-URA3* (CPY) or *PHO8-URA3* (PHO8) fusions. Growth in absence (−URA) or presence (+URA) of uracil was monitored on plates at 30°C. Growth in the absence of uracil indicates a defect in translocation into the ER. (**C**) Wildtype (BY4742) and the indicated mutant cells were grown to early log phase, cycloheximide added at time 0, and samples taken at the indicated chase times. Cells were lysed, proteins resolved by SDS PAGE and Sec62p and Pdi1p (as loading control) detected by immunoblotting.

Processing of the N-terminal methionine and the amino acid in position 2 determine which N-acetylation complex can modify a protein [23]. N-terminal processing and a serine in position 2 as in Sbh1p and Sec62p leads to N-acetylation by the NatA complex [23]. The gene encoding the enzymatically active subunit of NatA, *ARD1*, is not essential, but if N-acetylation played a subtle role in Sec complex function one would expect the *Δard1* strain to have growth defects exacerbated at higher or lower temperatures. We tested growth of a *Δard1* strain at 20°C, 30°C, and 37°C on solid media (Figure 7A) and by measuring growth curves in liquid media (not shown). We found that the *Δard1* strain had a longer generation time than the wildtype at all temperatures tested, and that this defect was more pronounced a low and high temperatures (Figure 7A). We then asked whether the growth defect was due to a protein translocation defect into the ER. To examine translocation we transformed the *Δard1* and the corresponding wildtype strain with reporter plasmids encoding fusions of the gene for a cotranslationally translocated protein, Pho8p, or a posttranslationally translocated protein, CPY, to *URA3*. In cells competent for translocation, the fusion proteins are efficiently translocated into the ER, and because the cells are chromosomally *ura3*, they cannot grow in the absence of uracil [24]. In cells defective in co- or posttranslational import into the ER, the corresponding substrate accumulates in the cytosol, and because the Ura3p part of the fusion protein is now in the appropriate location for its function, these cells can grow without uracil. As shown in Figure 7B, *Δard1* cells are fully competent for protein translocation into the ER in contrast to the *sec61-302* mutant which has a cotranslational import defect [24]. This is consistent with Forte et al. [5] who found no posttranslational import defect in *Δard1* cells by pulse-chase.

N-acetylation frequently enhances protein stability [2]. We asked whether Sec62p stability was compromised in the *Δard1* strain performing a cycloheximide chase experiment. Samples were taken up to 135 min, cells lysed, and Sec62p detected by immunoblotting. We found that Sec62p was stable in the *Δard1* and the wildtype strains for up to 135 min (Figure 7C). In contrast, mutant Sec62p in the *sec62-1* strain was unstable and rapidly turned over (Figure 7C). Expression levels of Sbh1p in the *Δard1* strain were also similar to wildtype (not shown). We conclude that the growth defect in the *Δard1* strain is not due to protein instability of Sec62p or Sbh1p or a protein translocation defect into the ER.

## Discussion

### N-acetylation of Sec complex subunits

Although N-acetylation is a frequent modification (50% of proteins in yeast) its function is not well understood so far [4]. For soluble secretory proteins it had been shown that N-acetylation interferes with their targeting to the ER [5]. In Sec complexes purified using an HA-tagged Sss1p, however, we found that in both Sec62p and Sbh1p the N-terminal methionine had been cleaved and the following serine N-acetylated (Figure 6A). In a genome-wide identification of N-acetylated proteins, Sec61p was also found to be modified in the same fashion (processing of the methionine, acetylation of S2) [25]. We likely missed the N-acetylated N-terminus of Sec61p because the trypsin digest prior to mass spectromety resulted in a very short N-terminal peptide. Effects of N-acetylation on protein stability, and on protein-protein interactions have been reported [2,3]. Mutation of the N-acetyl acceptor serine in position 2 in Sec62p had no effect on its function in protein translocation into the ER, however, and deletion of the enzymatically active subunit of the NatA complex, *Δard1*, did not affect Sec62p stability over a 135 min chase period (Figure 6B, C; Figure 7C). Mutation of S2 to tyrosine in Sec61p had no effect on protein stability at 30°C, but resulted in tunicamycin-sensitivity and temperature sensitivity at 37°C, without growth defects at lower temperatures (not shown). Sbh1p was also stably expressed in the *Δard1* strain (not shown), and the *Δard1* strain showed no gross defects in protein translocation into the ER either co- or posttranslationally (Figure 7B). Our data suggest that N-acetylation of Sec62p and Sbh1p is not important for protein stability, and not essential for their function in protein import into the ER. N-acetylation of Sec61p affects its function at high temperature (not shown), and will be investigated in more detail in the future. But since our *sec61-S2Y* mutant was temperature-sensitive only at 37°C, whereas the *Δard1* strain had growth defects at all temperatures tested (Figure 7A), it is likely that defects in other NatA substrates contribute to this *Δard1* phenotype.

As three transmembrane subunits of the Sec complex in the ER are N-acetylated, this argues against a general interference of this modification with protein targeting to the ER. In fact, Forte and colleagues [5] had only demonstrated an interference of N-acetylation with posttranslational targeting of soluble proteins to the yeast ER. For cotranslational targeting, the authors had found that these proteins, even if they contained an N-terminal sequence compatible with N-acetylation, were usually not modified [5]. The authors argued that competition of NatA and SRP for their ribosomal binding site would lead to presence of only one or the other near the exit tunnel of the large ribosomal subunit and thus in the presence of SRP NatA-mediated N-acetylation could not occur [5]. Here we find that two cotranslationally ER-targeted transmembrane proteins, Sec61p and Sec62p, are N-acetylated on a NatA consensus sequence (Figure 6 and [25]), suggesting that the binding of NatA and SRP to the nascent protein are limiting, not their binding to the ribosome. In soluble secretory proteins with N-terminal signal sequences SRP and NatA compete with each other for binding to the the nascent protein N-terminus. In transmembrane proteins like the Sec complex subunits the binding sites for NatA and SRP in the nascent protein are physically separated from each other, so both can bind. We also found the tail-anchored protein Sbh1p N-acetylated which shows that N-acetylation does not interfere with posttranslational ER-targeting by the GET machinery. Taken together our data and those in Forte et al. [5] suggest that N-acetylation only interferes with posttranslational ER targeting via the Sec63 complex.

### Phosphorylation of Sbh1p

Sbh1p is a small tail-anchored protein which interacts with multiple partners (see Introduction). It is the only subunit of the Sec61 channel that is non-essential, so it likely enhances speed of translocation or efficiency of targeting rather than forming a part of the channel proper. This view is supported by the crystal structure of the archaeal SecYEG complex, where SecG is associated with the periphery of the channel [26]. Sbh1p also is the only subunit of the Sec61 complex that has functions outside the protein translocation channel [17]. Protein-protein interactions can be regulated by covalent modifications such as N-acetylation and phosphorylation [1,3]. Phosphorylation in contrast to N-acetylation is reversible and therefore allows flexible regulation of specific interactions depending on specific circumstances.

The cytosolic domain of Sbh1p contains several predicted phosphorylation sites, and its mammalian orthologue, Sec61ß, had been shown to be phosphorylated, but the modified sites had not been identified, nor had the function of Sbh1p phosphorylation been investigated in detail [19]. The authors had demonstrated that in vitro phosphorylation of microsomal membranes enhances protein translocation into the ER, but since they had found three proteins involved in protein translocation phosphorylated (docking protein α, TRAP, and Sec61ß), the contribution of Sec61ß phosphorylation to the translocation enhancement remained unclear. When we mutated the predicted phosphorylation sites in Sbh1p individually or up to a quadruple combination (S21, T33, S38, S44) to alanine, we did not observe hypophosphorylation of Sbh1p, and even the quadruple mutant was still able to complement the temperature-sensitivity of a *Δsbh1Δsbh2* strain (Figure 2, and not shown). We therefore purified Sbh1p with Sec61 complexes using membranes from an *SSS1*-HA strain and subjected the purified protein to mass spectrometry analysis. We found that Sbh1p when it is part of the Sec61 complex is phosphorylated on a single site, T5 (Figure 3). Mutation of T5 to alanine, either alone or in combination with the only phosphorylatable residue not covered in our mass spectrometry analysis, S27, did again not result in detectable hypophosphorylation of Sbh1p in yeast cells, or in loss of function in translocation (Figure 4A, B). Mutation of T5 did result in a reduced signal on Western Blots, but because T5 is part of the peptide against which our antibody was raised, it remains unclear whether this reduction in signal is due to decreased interaction with the antibody, or to lower expression of T5A Sbh1p (Figure 4B, D). In a cycloheximide chase experiment, however, T5A Sbh1p was stable (Figure 4C).

Our data and a survey of the phosphoproteome data bases show that several sites can be phosphorylated in Sbh1p (Figure 4 and Table 1). With one exception [27], the investigators all found phosphorylation of multiple (up to three) sites of Sbh1p, but while there was overlap between sites found modified in an individual experiment, in no case was there identification of an identical set of phosphorylated residues [27-30] (Table 1). Modified phosphorylation sites in Sbh1p occurred in two clusters, one proximal to the N-terminus (S2, S3, T5, T12) and a second one closer to the transmembrane domain of Sbh1p (S35, S38) (Table 1). Mutation of S35, but not of S38, to alanine led to complete destabilization of Sbh1p in our hands, and the mutant was one of the few that was unable to complement growth of a *Δsbh1Δsbh2* strain (Table 1). By contrast, mutation of S35 to aspartate left the protein stable and complementation competent (Table 1).

**Table 1:** Potential & used phosphorylation sites in the cytosolic domain of Sbh1p

**Position**	**Mutant**	**Mutant complements Δsbh1Δsbh2**	**Position phosphorylated by mass spec**	**References**
*S2*	S2A *	✓	✓	[27]
*S3*	*S3A*	✓	✓	[28]
*T5*	*T5A*	✓	✓ ✓ ✓	our study; [28,29]
*T12*	T12A *	✓	✓	[28]
*S20*	S20A *	✓		
*S21*	*S21A*	✓		
*S27*	*S27A*	✓		
*T33*	*T33A*	✓		
*S35*	S35A *	X	✓ ✓	[29,30]
*S35*	*S35D*	✓		
*S38*	*S38A*	✓	✓ ✓	[29,30]
*Y43*	*Y43A*	X		
*Y43*	*Y43F*	✓		
*S44*	*S44A*	✓		
*T48*	T48A *	X		
*T48*	*T48D*	✓		

* extremely low expression levels.

Phosphorylation of S2 of Sbh1p was detected in one study [27]. This is unusual because S2 at the N-terminus of Sbh1p is also N-acetylated (Figure 3 and Figure 6), and there are no reports that we are aware of of N-termini that have both modifications. One possible explanation is that there are two populations of Sbh1p, one which is phosphorylated at S2, the other N-acetylated at S2. But mutation of S2 to alanine destabilized all Sbh1p dramatically and made it close to undetectable on Western Blots, although the low amount of residual protein was still able to complement the growth defect of *Δsbh1Δsbh2* yeast (not shown and Table 1). Alanine is less efficiently N-acetylated than serine, but the fact that Sbh1p is stable in a *Δard1* strain (not shown) suggests strongly that it is the lack of phosphorylation that is critical in the S2A mutant [4]. Sbh1p is a tail-anchored protein which is inserted into the ER membrane posttranslationally after dissociation from the ribosome, and the NatA complex is ribosome-associated [31,32]. Perhaps S2 is N-acetylated during biosynthesis and phosphorylation of the same residue early during biogenesis stabilizes the protein in a specific conformation that improves its interaction with chaperones or the insertion machinery in the ER membrane. If phosphorylation of S2 were important during biogenesis only, the phosphate might be removed once the protein is inserted into the ER membrane which would explain why in most studies S2 was not found phosphorylated (Table 1).

Phosphorylation of T5 of Sbh1p was detected in two phosphoproteome studies by mass spectrometry, but in both cases other sites were also found to be modified [28,29]. The principal difference between these studies and ours is that in the phosphoproteome analyses total cellular Sbh1p was detected, so when multiple sites were found phosphorylated it is not clear whether these were in the same molecule or in different populations of Sbh1p. In our work, we identified T5P Sbh1p in Sec61 complexes that were either free or associated with the Sec63 complex (Figure 3). We have no information on the phosphorylation status of Sbh1p in ribosome-associated Sec61 complexes, of exocyst-associated and of free Sbh1p. Comparison of our own and the phosphoproteome data suggest that the phosphorylation state of and sites used in Sbh1p may differ depending on its interaction partners, and/or on growth conditions.

The kinases modifying Sbh1p may include proline directed kinases like Cdc28 for T5, and perhaps casein kinase 2 (CK2) for the membrane proximal residues S35 and S38 [1]. The latter do not fulfil the consensus sequence for CK2 modification, but CK2 has been shown to phosphorylate other residues in non-consensus contexts and CK2 modifies Sec63p, which would bring it into close proximity with Sbh1p in the Sec complex [1,20]. In our mutagenesis studies, we never saw hypophosphorylation attributable to mutation of a specific residue, even when we mutated all available serines and threonines in the cytosolic domain of Sbh1p individually, or when we mutated several sites in combination (Figure 2, Figure 4; Table 1). Kinases in yeast tend to be promiscuous, however, and if their actual target residue is missing they can phosphorylate other serine or threonine residues in the vicinity [33]. Promiscuous phosphorylation may therefore be part of the explanation for the lack of hypophosphorylation in the T5A mutant.

Mammalian Sec61ß (mouse and human) has also been found in several phosphoproteome analyses to be modified on multiple sites, but the pattern of phosphorylation of Sec61ß was different from Sbh1p (all residues highlighted in the first line of Figure 1B, with the exception of S43 & T48; ProSite). Only some these these sites are conserved in yeast including T5 (yeast numbering: T5, S21, T33), and of these only T5 was found phosphorylated in yeast (Table 1). When Gruss and colleagues investigated the phosphorylation of mammalian Sec61ß, they did not identify the modified residues, but found after tryptic digest and phosphopeptide mapping that a single peptide was predominantly phosphorylated (O. Gruss, personal communication). From the distribution of trypsin cleavage sites and phosphorylated residues this peptide is almost certainly identical to the N-terminal 15 amino acids of Sec61ß which includes T5 and two serine residues which were found to be phosphorylated in phospho-proteome studies (Figure 1B; ProSite).

Phosphorylation of the cellular pool of Sbh1p was dynamic (Figure 5A) in contrast to phosphorylation of Sec63p (Figure 5A), thus phosphorylation of Sbh1p has the potential to regulate its interactions with its various partners. Phosphorylation at specific sites may either enhance a specific interaction or prevent it [34]. The phosphate that we found on T5 may prevent binding of the Sbh1p cytosolic domain to the ribosome when the Sec61 complex is associated with the Sec63 complex. Based on crosslinking experiments to nascent chains the N-terminus of Sbh1p can reach into the peptide exit tunnel of the large ribosomal subunit [13]. Phosporylation of T5, which adds bulk and two negative charges to a position fixed by two flanking prolines, will almost certainly reduce access of the Sbh1p N-terminus to the peptide exit tunnel and may at the same time reduce affinity of Sbh1p and the Sec61 complex for the ribosome. Kinases frequently are dependent on each other [1], so phosphorylation of T5 may enhance phosphorylation of additional sites in Sbh1p when it has dissociated from the Sec63 complex which may signal to the SRP receptor that this translocon is unoccupied and available to receive a new nascent chain (similar to Sbh2p in [10]). Interaction with the SRP receptor may then trigger dephosphorylation of T5 and allow Sbh1p to bind to the ribosome again. Other scenarios in which T5 phosphorylation regulates interaction of Sbh1p with the Sec63 complex, promotes interaction of Sbh1p with the Sec61 complex, or prevents interaction with the exocyst are also plausible. In Sbh2p, which forms a strictly cotranslational translocon with the Sec61p homologue Ssh1p and does not interact with the exocyst, the threonine in position 5 is not conserved (Figure 4D). T5 phosphorylation is therefore likely to have an effect on interactions that are specific to Sbh1p, i.e. interaction with Sec61p, with the Sec63 complex or with the exocyst.

The N-terminal cytosolic domain of Sbh1p is predicted to be largely unstructured and indeed in the crystal structure of the archaeal channel the SecG cytosolic domain is not visible [26]. The Sbh1p cytosolic domain also contains a relatively large number of basic amino acids most of which are conserved between yeast and dog (Figure 1B). One conceivable effect of T5 phosphorylation is a folding back of the Sbh1p N-terminus and interaction of the negatively charged phospho-T5 with one of the basic patches more proximal to the membrane (amino acids 15–17 KRK, or 30/31 KK). This phosphorylation-induced structure may then promote or prevent interaction with known Sbh1p binding partners, and/or with additional kinases. The prolines flanking T5 may also be subject to cis-trans isomerization by phosphate-directed prolyl isomerases like Pin1p which may help the phosphorylated Sbh1p N-terminus to acquire its functionally relevant structure [22].

The phosphorylation site at T5 almost certainly has an interesting role, as both the site and its flanking prolines are conserved between *S. cerevisiae* and mammals (Figure 1B), but not Xenopus, fish, invertebrate metazoa, or other yeasts (Figure 5B). Kinases evolved largely after the development of metazoa, so phosphorylation sites, kinases, and the actual use of the phosphorylation sites are generally poorly conserved between yeast and mammals [1,34,35]. In two birds, chicken (*Gallus gallus*) and zebrafinch (*Taeriopygia guttata*), the residues surrounding the T5 phospho-acceptor site are conserved (Figure 5B) and in chicken, T5 is replaced by a phosphorylatable serine, whereas in zebrafinch T5 is replaced by asparagine (Figure 5B). Altogether the data suggest that the N-terminus of Sec61ß proteins is evolving rapidly, and the fact that the PTP motif occurs in yeast and again in some birds and all mammals suggests a case of convergent evolution.

## Conclusions

As three subunits of the ER-resident Sec complex are N-acetylated, the modification per se is unlikely to interfere with targeting to the ER. That N-acetylation can occur on two cotranslationally targeted transmembrane proteins, Sec61p and Sec62p, but not on cotranslationally targeted soluble proteins [5], suggests that in signal-sequence bearing proteins binding of NatA and SRP to the nascent protein are limiting, not their binding to the ribosome. Since we also found the tail-anchored protein Sbh1p N-acetylated, our data and those in Forte et al. [5] demonstrate that N-acetylation exclusively interferes with posttranslational ER targeting via the Sec63 complex.

Our phosphosite analysis of Sbh1p suggests that several Sbh1p populations exist in the cell which are phosphorylated at different sites in different combinations. The phosphate on T5 of Sbh1p must play a particularly important Sbh1p-specific role since the site evolved twice independently, in *S. cerevisiae* and in mammals, and is not present in Sbh2p. Phosphorylation is a reversible modification known to affect protein-protein interactions, thus it is likely that the phosphorylation status of an individual Sbh1p molecule determines its interaction partners, and vice versa. In the future we will therefore investigate the phosphorylation status of exocyst- and ribosome-associated Sbh1p by mass spectrometry, and subsequently make the readout for functional analysis of specific phospho-site mutations specific to the complex in which they were found to be modified.

## Methods

### Yeast strains

NY179 (*SBH1 SBH2 MAT* a *leu2-3,112 ura3-52*), H3223 (MAT a *KanMx::sbh1 leu2-3,112 ura3-52 GAL*^*+*^), H3203 (MAT a *HphMx::sbh2 leu2-3,112 ura3-52 GAL*^*+*^), H3231 (MAT a *KanMx::sbh1 HphMx::sbh2 leu2-3,112 ura3-52 GAL*^*+*^) were gifts from Jussi Jäntti and used to characterize Sbh1p phosphorylation sites [7,8]. KRY739 (*SSS1-HA::TRP1 ssh1Δ::ADE2*^*+*^) was a gift from Kai-Uwe Kalies and used to purify Sec complexes. BY4742 (*MAT* a *his3-1 leu2 lys2 ura3 can1-100*), BY17299 *(Δdoa10::KanMX6* in BY4742), and BY10976 *(Δard1::KanMX6* in BY4742) were from the Euroscarf collection and a gift from Manfred Schmitt. RSY1132 (*MAT* a *leu2,3-112 trp1-1Δ ura3-52 sec61-3*) [36], RSY1294 (*MAT* a *leu2,3-112 can1-100 leu2-3,112 his3-11,15 trp1-1 ura3-1 ade2-1psec61-32*) [37], RSY529 (*MAT* a *leu2,3-112 his4-619 ura3-52 sec62-1*) [38] were gifts from Randy Schekman. KRY712 (BMA38a *MAT a his3-Δ200 leu2-3.112 ura3-1 trp1-Δ1 ade2-1 can1-100* [pCEN-*LEU2sec61-302*) was a control for the translocation reporter constructs [24].

### Plasmids and site-directed mutagenesis

*SBH1* cloned into pCR2.1-TOPO plasmid was kindly provided by Jussi Jäntti (Helsinki University, Finland). The QuickChange Site-Directed Mutagenesis Kit (Stratagene, UK) was used to introduce single or multiple base mutations in *SBH1.* Substitutions were verified by sequencing. Mutated *sbh1* was excised from pCR2.1-TOPO plasmids using EcoRV/BamHI, and subcloned into pRS305, pRS415 and pRS424. The second amino acid of Sec62p was changed from serine to tyrosine as above in *SEC62* in pUC19. After verifying the sequence mutant *sec62-S2Y* was subcloned using SacI/HindIII into pRS315. Plasmids with *PHO8-URA3* and *PRC1-URA3* are described in [24].

### Phosphate labelling of microsomal membranes

Microsomes were prepared from *SBH1/2* wildtype or mutant cells as in [37]. Labelling reactions contained 5eq membranes in B88 with 0.1 μM okadaic acid and calyculin A (Calbiochem, UK), 5 μM GTP, 2 mM CaCl2, 40 μCi γ-^32^P]ATP (Amersham Biosciences, UK). Reactions were incubated at 30°C for 30 min and okadaic acid and calyculin A (500 nM) in B88 were added prior to sedimentation at 14,100 x g at 4°C for 5 min. Membranes were lysed in 50 μl 2% SDS, incubated at 65°C for 10 min, Sbh proteins immunoprecipitated with anti-Sbh1p serum raised by us against the first 18 amino acids of Sbh1p. Samples were analyzed by SDS-PAGE on 15% gels and autoradiography.

### Phosphate labelling of intact cells

Yeast grown in YNBD –Leu media to OD_600_ = 1 were pelleted, washed once with YNBD –Leu –PO_4_ media, and resuspended in phosphate free medium to OD_600_ = 1.5. Cells were labelled with [^32^P]PO_4_ (75 μCi per sample, Amersham Biosciences UK) for 1 Â½ hr at 30°C with gentle agitation. Equal volumes of TCA (25%) were used to precipitate cells for 20 min on ice; pellets were washed twice in ice-cold acetone before air-drying. Cells were lysed with 100 μl lysis buffer (50 mM Tri-Cl pH 7.5, 1 mM EDTA, 1% SDS, 6 M urea) and an equal volume of glass beads (acid washed, Sigma) by two cycles of 1 min vortexing and 1 and 10 min heating at 65°C respectively. The lysate was diluted in IP buffer containing 100 nM okadaic acid and calyculin A and 1 mM AEBSF (Calbiochem UK). Cell debris were sedimented by centrifugation prior to immunoprecipitation of Sbh1p and Sec63p. For pulse-chase experiments cells were labelled for 10 min as above, the samples (duplicates) were washed in phosphate-free media (YNBD –Leu –PO_4_) and resuspended to the original OD_600_ (1.5) with YNBD –Leu media and chased for 5, 15, 30, 60 min.

### Cycloheximide chase

For Sbh1p cells grown in YNBD -Leu to OD_600_ = 1.5 were washed in YNB –Leu, resuspended in that medium to the same OD and incubated at 30°C for 30 min in a shaking incubator. Cycloheximide (Roche UK) was added to 50 μg/ml [39] and the cells incubated for up to 90 min. The reaction was stopped by addition of equal volumes of cold TCA (25%) for 20 min. Cells were lysed as above and Sbh1p detected by immunoblotting. For Sec62p cells were grown in YPD to OD_600_ = 1.0, cycloheximide added to 200 μg/ml, samples taken at the indicated times, and cells lysed by bead-beating. After gel electrophoresis, Sec62p was detected by immunoblotting.

### Sec complex purification for mass spectrometry

7,500eq of microsomal membranes from the SSS1-HA strain in B88 were and lysed in 2.5% digitonin buffer [50 mM HEPES-KOH pH: 7.4, 400 mM potassium acetate, 8 mM magnesium acetate, 100 nM okadaic acid & calyculin A (Calbiochem UK),1 x protease inhibitor cocktail (Roche UK), 10% glycerol, 2 mM DTT, 2.5% digitonin (high purity, Calbiochem UK)] for 30 min at 4°C with gentle agitation. Solubilised membranes were sedimented (70,000 rpm, TLA-100.3 Beckman rotor) for 1 h at 4°C; the supernatant was diluted with the same buffer without digitonin, to 1% digitonin. Monoclonal anti-HA agarose conjugate (Sigma UK) was washed 3x with TBS and twice with 10 ml digitonin buffer, then lysate was added for overnight binding at 4°C with rotation. The resin was washed twice with 10 ml of solubilization buffer with 1% digitonin not containing glycerol, 3x with 10 ml TBS at 4°C. Bound proteins were eluted at room temperature with 4 ml of 200 mM Glycine-HCl pH: 2.5, TCA-precipitated and analysed by 12% SDS-PAGE and Coomassie Brilliant Blue (BioRad UK) staining. The band corresponding to Sbh1p was excised and stored in 10% Methanol before trypsin-digest and mass spectrometry analysis. Purification for detection of N-acetylation was done as above, but without phosphatase inhibitors. Bands were excised, digested with trypsin overnight, and resulting peptides analyzed by MALDI-TOF/TOF mass spectrometry on a MALDI 4800 TOF/TOF analyzer (Applied Biosystems). Tryptic peptide mass fingerprints were measured in positive reflector mode with subsequent MSMS fragmentation. Combined MS & MSMS data were identified using the NCBInr protein data base.

## Competing interests

All authors declare that they have no competing interest.

## Authors’ contributions

CS carried out the genetic & biochemical analysis of the phosphosites in Sbh1p. NZ purified the Sec complexes for identification of phosho-T5 in Sbh1p. KH and EH performed the mass spectrometry of Sec complex subunits that identified N-acetylated proteins. MCK and TT performed the analyses of N-acetylated Sec complex subunits. MNJS participated in the design of the phosphorylation analysis, co-supervised CS and contributed to the writing of the manuscript. KR conceived the study, participated in the design and coordinated the work, and wrote the manuscript. All authors read and approved of the final manuscript.
